# Effect of Day Length on Growth and Gonadal Development in Meishan Male Pigs

**DOI:** 10.3390/ani14060876

**Published:** 2024-03-13

**Authors:** Naisheng Lu, Hao Yuan, Xueyuan Jiang, Hulong Lei, Wen Yao, Peng Jia, Dong Xia

**Affiliations:** 1 Key Laboratory of Livestock and Poultry Resources (Pig) Evaluation and Utilization of Ministry of Agriculture and Rural Affairs, Shanghai Engineering Research Center of Breeding Pig, Institute of Animal Husbandry and Veterinary Science, Shanghai Academy of Agricultural Sciences, Shanghai 201106, China; lunaisheng@saas.sh.cn (N.L.); yhaonjau@163.com (H.Y.); jiangxueyuan@saas.sh.cn (X.J.); leihulong@saas.sh.cn (H.L.); jiapeng@saas.sh.cn (P.J.); 2 College of Animal Science & Technology, Nanjing Agricultural University, Nanjing 210095, China; yaowen67jp@njau.edu.cn

**Keywords:** day length, male pigs, behavior, testicular size, testosterone, melatonin receptors, sex hormone synthesis proteins

## Abstract

**Simple Summary:**

Day length is a critical environmental factor for gonadal development in animals. However, related studies on male pigs are limited. The present study investigated the effects of different day lengths on behavior changes, growth parameters, testicular size, testosterone secretion, steroidogenesis proteins, and melatonin receptors in Meishan male pigs. The results indicated that a long day length (LDL, 14 h light/10 h dark, 14L/10D) could increase the lying time, decrease the exploring time, and increase the body height, chest circumference, testicular length, testicular weight, crude protein digestibility, and fecal testosterone and cortisol contents compared to a short day length (SDL, 10L/14D) in Meishan male pigs, accompanied by the changes in sex hormone synthesis proteins and melatonin receptor 1b (MT2), but with no change in melatonin receptor 1a (MT1). This suggests that the effect of day length on growth and gonadal development in male pigs may be conducted via MT2 and influence steroid synthesis and secretion in the testis, and proper day length should be provided in male pig breeding.

**Abstract:**

Day length is a critical environmental factor for regulating animal growth and development. This study aimed to investigate the effects of different day lengths on the developmental changes of growth parameters, testicular sizes, testosterone secretion in Meishan male pigs, and steroidogenesis proteins and melatonin receptors. Fourteen Meishan male pigs (10 weeks (wks) of age) with the same parity, paired in litter and body weight (BW), were evenly allocated into a short-day-length group (SDL, 10 light/14 dark) and long-day-length group (LDL, 14 light/10 dark). After 12 wks of the experiment, the LDL-treated boars had more lying time and less exploring time. The LDL treatment led to significant increases in body height, chest circumference, testicular length, testicular weight, crude protein digestibility, and fecal testosterone at the 10th and 12th wks of the experiment, and cortisol at the 10th wk, compared to the SDL treatment, with no differences in the final BW, testicular width, and epididymis weight. Furthermore, the LDL treatment significantly increased the protein levels of melatonin receptor 1b (MT2), aromatase (CYP19), and steroidogenic factor 1 (SF1) in the testis, with no differences in the protein levels of melatonin receptor 1a (MT1), steroidogenic acute regulatory (StAR), 3β-hydroxysteroid dehydrogenase (3β-HSD), and cholesterol side-chain cleavage enzyme (P450scc). The present study suggests that day length has an effect on the growth and gonadal development in male pigs maybe via MT2 and influences steroid synthesis and secretion in the testis. Therefore, proper day length should be considered in male pig breeding.

## 1. Introduction

Daily lighting time (day length) is one of the critical environmental factors for growth and gonad development in animals. Research on boars indicated that a treatment of 16 h (h) day length (16 light/8 dark, 16L/8D) increased the serum testosterone concentrations compared to the 8L/16D treatment [1]. The 15L/9D treatment improved sperm parameters in boars compared to those exposed to a natural autumn light regime [2]. Interestingly, 16L/8D-treated nursery piglets had a higher body weight gain (BWG), lower cortisol levels in the blood, and a better immune status than 8L/16D-treated piglets [3]. A treatment of 23L/1D increased the feed intake and average daily gain (ADG) compared to 8L/16D-treated piglets [4]. In addition, the 18L/6D regime promoted the development of gonads and ovarian function in gilts compared to complete continuous darkness or natural winter day length [5]. Together, this indicates that a daily lighting time of more than 12 h could improve the growth and development of pigs; however, the underlying mechanisms of this remain unclear.

In males, light signals are transmitted via the suprachiasmatic nucleus (SCN) to the pineal gland and drive the circadian rhythm secretion of melatonin [6], which is conducted via melatonin receptors and influences the activity of the hypothalamus–pituitary–testis (HPT) axis and regulates the production of sex steroids [7,8]. Researchers observed melatonin receptor expression in the testis [9,10], and the silencing of melatonin receptor reduced adenyl cyclase (cAMP) activity and decreased testosterone secretion [11]. The testis is the target organ of HPT, the site of testosterone synthesis, secretion, and spermatogenesis [12]. Testosterone is a critical steroid hormone required for the development of male characteristics and also supports the physiology of the male reproductive system [13]. The synthesis and secretion of testosterone involve several steroidogenesis proteins, including steroidogenic factor 1 (SF1), steroidogenic acute regulatory (StAR) [14], cholesterol side-chain cleavage enzyme (p450scc), and 3β-hydroxysteroid dehydrogenase (3β-HSD) [15]. The protein levels of SF1 increased with the testicular development, and the SF1 expression of testes was positively correlated with the serum testosterone level [16]. The inactivation of SF1 in Leydig cells caused a reduction in testosterone production [17]. As rats’ age increased, the reduction in testosterone secretion from Leydig cells was correlated with a decrease in the abundance of StAR, p450scc mRNA, and protein [18]. The increased mRNA levels of StAR, p450scc, and 3β-HSD parallel the improvement in testosterone production in vivo adult rat testis and in vitro immature rat Leydig cells [19]. However, there are limited reports on the effect of day length on melatonin receptors and steroidogenesis proteins in the testis.

Local pig breeding farms in Shanghai usually have a day length of 10L/14D in the wintertime and 14L/10D in the summertime. However, which day length is suitable for the development of male pigs remains to be discovered. Therefore, this study investigated the impacts of different day lengths on the behavior changes, and the developmental changes in body sizes, body weight (BW), testicular size, and testosterone secretion, in Meishan male pigs, which are a local Chinese breed originating from the Lake Taihu region west of Shanghai and are well known for their high fertility [20]. In addition, the present study analyzed the levels of melatonin receptors and steroidogenic proteins including SF1, StAR, 3β-HSD, P450scc, and aromatase (CYP19) in the testis to further clarify the underlying mechanism of this.

## 2. Materials and Methods

### 2.1. Animals and Sampling

The present study took 10L/14D (light from 08:00 to 18:00) as the short-day-length (SDL) treatment, and 14L/10D (light from 08:00 to 22:00) as the long-day-length (LDL) treatment. Meishan male pigs start puberty as early as 75 days of age, and reach sexual maturity at approximately 5 months of age [21,22]. This study focused on the developmental changes in Meishan male pigs from puberty to sexual maturity. Therefore, the starting age for Meishan male pigs in the experiment was 10 weeks (wks) old (pre-puberty), the experiment duration was 12 wks, and the ending age was 22 wks old. Fourteen Meishan male pigs (10 wks of age) from three litters with the same parity, paired in litter and BW, were allocated into SDL group and LDL group, with seven boars grouped in a pen (n = 7 per group). During the 12 wks of the experiment, all pigs lived in environmentally controlled rooms. The light intensity was maintained at 50 lux (Digital Lux Meter, TES-1330A, TES Electrical Electronic, Taiwan, China) at pigs’ eye level when the light-emitting diode (LED) light (wavelength, 450 nm) hung 2 m above the pen floor automatically turned on, and pigs were fed the same commercial boar diet and nipple water ad libitum.

The BW of pigs was measured individually at the start and end of the experiment. Fresh stools were separately collected via rectal massage without contamination with the barn floor every 2 wks, then mixed homogeneously and individually over ice and stored at −20 °C until the analysis of hormones and apparent nutrient digestibility was conducted. At the end of the experiment, the final BW, BWG, body height, body length, and chest circumference were measured.

When experimental animals were paired according to their litter and BW, the animals had identical genetic and breeding backgrounds, meaning that fewer individuals could have statistical power [23]. Generally, the use of from three to four replicates for each group in the slaughter trial and Western blot analysis is acceptable [24,25]. Therefore, four pair boars from two groups were randomly selected and sacrificed with CO_2_ gas [26], and the testis and epididymis weight, length, and width were recorded. Meanwhile, the testis sample was frozen in liquid nitrogen and stored at −80 °C until Western blot analysis.

### 2.2. Apparent Total Tract Digestibility

The experimental feed and feces samples were dried at 105 °C until they reached a constant weight to determine the dry matter (DM), and then milled through a 40-mesh screen. Crude protein (CP) was calculated through the determination of the nitrogen (N) content by the Kjeldahl method according to the Association of Official Analytical Chemists (AOAC, 2005). The acid-insoluble ash (AIA) was taken as a marker for measurement of the apparent total tract digestibility (ATTD) of DM and CP, as previously described [27].

### 2.3. Behavior Recordings

The boar behavior was monitored via infrared video camera (DS-7608N-E2/8P, Hikvision, Hangzhou, China), which was hanging on the roof, with a view of the entire pen. The behavior of each boar during the 24 periods of at 12th wks was recorded and stored. Through the video playback, the frequency and lasting time of each behavior, including drinking, feeding, lying, mounting, and exploring, were observed and registered by a trained observer according to the behavior definitions presented in Table 1 [28,29].

### 2.4. Determination of Fecal Steroid Hormones Concentration

Compared to blood sampling, fecal sample collection is non-invasive, leading to less interference from acute stress [30]. Furthermore, the fecal steroid hormone concentration can represent systemic steroid hormone synthesis and secretion [31]. Moreover, fecal steroid hormone levels display smooth short-term fluctuations and diurnal variation. Fecal steroid hormone measures can improve the ability to distinguish between regular pulsatile changes and genuine physiological responses to external events [32]. Notably, the fecal samples represent a cumulative measure of hormone release over a more extended period, rather than a ‘snapshot’ or point sample, as in plasma samples [33]. Fecal hormone analysis has already been widely applied in wild species, particularly wild boar [34,35]. Therefore, the present study determined the fecal steroid hormones that were used to investigate sexual development following the previous protocol [36]. In brief, fecal samples were freeze-dried for 72 h, the sample weight before and after freeze-drying was recorded for the calculation of stool water content, and then 0.10 g dried fecal powder was extracted in 3 mL of 80% aqueous methanol by vortex for 15 min. Following extraction, the suspension was centrifuged, and the supernatant was recovered for hormone measurement.

The testosterone and cortisol content were measured using commercial EIA kits (Cayman (Ann Arbor, MI, USA), testosterone, Item No. 582701, intra-assay CV < 6.0%, inter-assay CV < 7.2%, sensitivity is 6 pg/mL; cortisol, Item No. 500370, Intra-assay CV < 9.5%, Inter-assay CV < 10.4%, sensitivity is 110 pg/mL) according to the manufacturer’s instructions. All samples were run in triplicate.

### 2.5. Western Blot Assay

The testis samples were fully homogenized and lysed in radioimmunoprecipitation assay (RIPA) lysis buffer containing 1 mM phenylmethanesulfonyl fluoride (PMSF) with electric homogenizer. The total protein concentration was determined using BCA protein assays. Then, a 15 μg protein sample (heat denaturation) was loaded and separated on 10% SDS-PAGE gels, and transferred to PVDF membranes, then blocked with 5% (*w*/*v*) non-fat dry milk (B&D, Sparks, MD, USA) in TBS containing tween 20 (TBST) for 2 h at room temperature. Subsequently, the PVDF membranes were incubated with the indicated antibodies. We selected the marker proteins in the principal pathways of steroid hormone synthesis and melatonin receptors [37,38], including rabbit anti-β-actin (1:1000, Bioss, Beijing, China, bs-0061R), rabbit anti-melatonin receptor 1a (MT1) (1:1000, Bioss, bs-0027R), rabbit anti-melatonin receptor 1b (MT2) (1:1000, Bioss, bs-0963R), rabbit anti-SF1 (1:500, Santa, Dallas, Texas, USA, sc-28740), StAR (1:500, Santa, sc-25806), 3β-HSD (1:500, Santa, sc-30820), P450scc (1:500, Santa, sc-18040), and CYP19 (1:1000, Santa, sc-374176). The cross-reactivity information for every antibody is provided in the Supplementary Materials File: Table S1. Primary antibody incubation was performed at 4 °C overnight, followed by incubations with the appropriate secondary antibody (1:5000, Bioss) for 1 h at room temperature. The protein bands were captured by Tanon-5200 Chemiluminescent Imaging System (Tanon Science & Technology, Shanghai, China), the intensity of blots was analyzed using ImageJ software (ver. 1.51j8, National Institutes of Health, Bethesda, MD, USA), and the protein levels were normalized to β-actin protein.

### 2.6. Statistical Analysis

Data organization and scientific graphing were performed using Microsoft Excel Office Professional Plus 2013 (Redmond, WA, USA). The distribution and the equality of variances were verified for all data. A data analysis was performed using 18.0 for Windows 10 (SPSS Inc., Chicago, IL, USA). Paired-samples T-test was used to test the significant differences in SDL and LDL groups. Further, the correlations between the testosterone content of feces, testicular weight, testicular length, and testicular width were assessed by Pearson’s correlation test using SPSS. All the data are expressed as means ± standard error of the mean (SEM). All the differences were considered significant at *p* < 0.05 for all tests.

## 3. Results

### 3.1. Growth Performance, Gonad Development, and Feed Digestibility

As shown in Table 2, at the end of the experiment (12 wks), compared to the SDL-treated boars, the LDL-treated boars showed an increase in body height (*p* = 0.025), chest circumference (*p* = 0.011), testicular length (*p* = 0.033), and testicular weight (*p* = 0.034). There were no differences in the body length, testicular width, and epididymis weight between LDL and SDL groups.

In addition, the LDL treatment significantly increased the ATTD of CP (*p* = 0.028) (Table 3). However, there were no differences in the final BW, BWG, and ATTD of DM between the LDL and SDL groups (Table 3).

### 3.2. Behaviors

As shown in Table 4, the LDL-treated boars had less exploring time (*p* = 0.039) and more lying time (*p* = 0.002) than the SDL-treated boars at 12 wks of the experiment. There were no differences in the frequency of mounting, drinking, and feeding between the LDL and SDL treatments at 12 wks of the experiment (Table 4).

### 3.3. Testosterone and Cortisol Content

**Figure 1:**
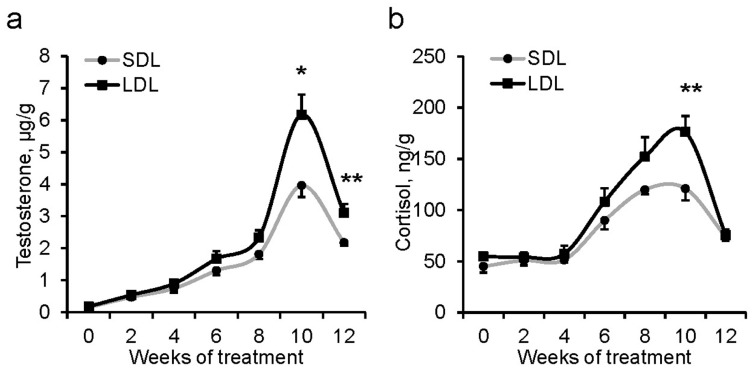
The content of testosterone and cortisol in feces: (**a**) testosterone, (**b**) cortisol. Data are expressed as the mean ± SEM (n = 7), * indicates significant difference between the short-day-length (SDL, 10L:14D) group and long-day-length (LDL, 14L:10D) group at different weeks of treatment, * *p* < 0.05, ** *p* < 0.01.

As shown in Figure 1, the fecal testosterone and cortisol contents had a similar developmental tendency in both the LDL and SDL treatments. The fecal testosterone and cortisol levels gradually increased during the first 8 wks of the experiment, peaked in the 10th wk, and decreased after that. There was no difference in the fecal testosterone and cortisol contents during the first 8 wks of the investigation between the LDL and SDL treatments. However, the LDL-treated boars had significantly higher fecal testosterone content at the 10th wk and 12th wk (*p* = 0.045, 0.008, Figure 1a), and higher fecal cortisol content at the 10th wk (*p* = 0.010, Figure 1b), than the SDL-treated boars.

### 3.4. Correlation Analysis

The correlation analysis showed that the testosterone content was positively correlated with the testicular length (r = 0.707, *p* = 0.049), and there was a positive correlation between the testosterone content and testicular weight (r = 0.691, *p* = 0.058), and testicular weight and testicular width (r = 0.691, *p* = 0.058), although this did not reach the significance level of *p* < 0.05 (Table 5).

### 3.5. The Protein Levels of Melatonin Receptors and Steroid Hormone Synthesis

**Figure 2:**
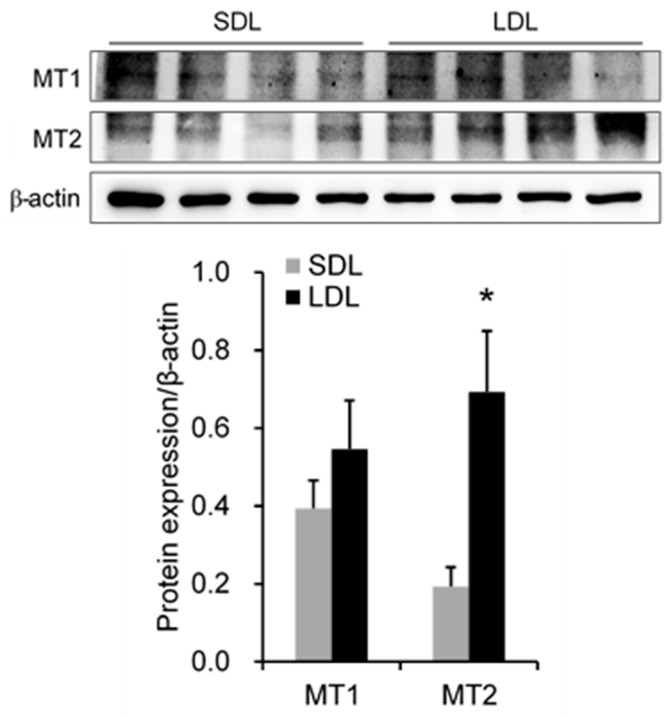
The protein levels of melatonin receptors. MT1, melatonin receptor 1a; MT2, melatonin receptor 1b. Data are expressed as the mean ± SEM (n = 4), * indicates significant difference between the short-day-length (SDL, 10L:14D) group and long-day-length (LDL, 14L:10D) group, * *p* < 0.05.

**Figure 3:**
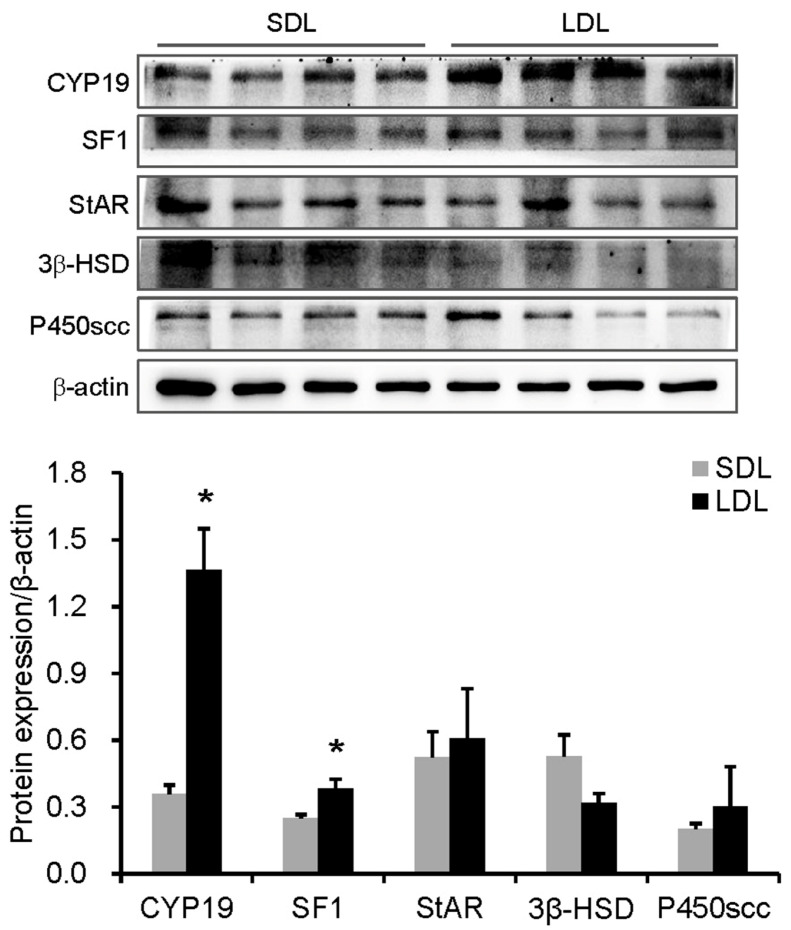
The protein levels related to steroid hormone synthesis. CYP19, aromatase; SF1, steroidogenic factor 1; StAR, steroidogenic acute regulatory; 3β-HSD, 3β-hydroxysteroid dehydrogenase; P450scc, cholesterol side-chain cleavage enzyme. Data are expressed as the mean ± SEM (n = 4), * indicates significant difference between the short-day-length (SDL, 10L:14D) group and long-day-length (LDL, 14L:10D) group, * *p* < 0.05.

Western blot assay indicated that the LDL treatment significantly increased the protein levels of MT2 (*p* = 0.026) (Figure 2), CYP19 (*p* = 0.019), and SF1 (*p* = 0.023) (Figure 3) compared to SDL treatment in the testis of boars. However, there were no differences in the proteins of MT1, StAR, 3β-HSD, and P450scc between the LDL and SDL treatments.

## 4. Discussion

Previous studies reported that the body weight, paired testis weight, and epididymis weight of Meishan male pigs continuously increase from birth to 16 wks of age, and the blood testosterone concentration gradually increases during the first 15 wks of age and declines to a plateau at 16 wks of age during pubertal development [21,39]. Interestingly, the present study observed a similar developmental tendency in the fecal testosterone concentration of Meishan male pigs, which gradually increased during the first 8 wks of the experiment, peaked at the 10th wk of the experiment, and declined at the 12th wk. This suggests that the concentration of fecal steroid hormones can be used to represent the systemic steroid hormones synthesis and secretion during periodic investigations [31].

Testosterone is synthesized and secreted from the testis, and in turn, testosterone regulates testis development, spermatogenesis, and germ cells’ apoptosis [40]. The developmental increase in testicular volume was accompanied by changes in blood testosterone content [12]. Male pigs with larger testes usually have higher sperm numbers, superior mating efficiency [41,42,43], and higher testosterone levels [44]. Consistently, the LDL-treated boars in the present study had a more considerable testicular length, weight, and fecal testosterone levels at the 10th and 12th wks of the experiment than those in the SDL-treated boars, and the fecal testosterone content positively correlated with the testicular length. This indicates that the LDL treatment may benefit testis growth and testosterone secretion in Meishan male pigs.

The present experiment showed a similar development in the fecal cortisol level as was found in the testosterone, and the LDL-treated boars had higher fecal cortisol levels than the SDL-treated boars at the 10th wk of the experiment. Research has indicated that testosterone can mutually modulate one another, with cortisol regulating energy metabolism [45] and behavioral events [46]. Cortisol is a steroid hormone and the end product of the hypothalamic–pituitary–adrenal (HPA) axis and is synthesized from cholesterol [46]. It can regulate the male reproductive function [47] and act on various tissues to elicit the energy metabolism and maintain homeostasis [48,49]. For many individuals, competition and status challenges result in increases in cortisol as well as testosterone [50,51]. Dominant male wolves had higher fecal testosterone and glucocorticoid metabolite concentrations than subordinate wolves [52]. Exogenous testosterone treatment increased the salivary cortisol concentration in dominant men in response to stress [53]. The associations between testosterone and behavior such as risk-taking or aggressive behavior are strengthened at high cortisol levels [54,55]. Furthermore, testosterone is an essential hormone for bone gain and maintenance and function in males [56,57]. In the present study, the LDL-treatment boars had a higher fecal testosterone content, increased body height, greater chest circumference, and a higher ATTD of CP. These results indicated that the LDL-treatment boars had superior feed utilization and may use more energy, which could account for the increased growth and reproductive activity that were achieved through the regulatory effect of testosterone and cortisol.

In addition, the improved ATTD of CP coincided with several behavioral indicators. At the 12 wks of the experiment, the LDL-treated boars had higher ATTD of CP than the SDL-treated boars; accordingly, the LDL-treated boars spent more time lying and less time exploring. These results are consistent with Holt et al. (2006), who found that the pigs with higher CP digestibility spent more time lying and less time exploring [58]. Lee et al. (2020) observed that the experimental group had a higher ATTD of CP as well as an increased gain-to-feed ratio in the growing-finishing pigs [59]. The changes in behaviors are related to day length. Martelli et al. (2005) reported that the pigs at the end of the 14L/10D regime spent more time resting and less time on abnormal behaviors compared to the 8L/16D-treated pigs [60]. The 16L/8D treatment decreased the time pigs spent exploring the floor and increased the resting time (lateral and sternal recumbency) compared to the 8L/16D male pigs [61]. Consistently, here, the LDL-treated boars spent more time lying and less time exploring at the 12th wk of the experiment. The LDL treatment induced more time lying and less exploring, which may account for the higher ATTD of CP.

However, research data about the effect of natural light regimes on growth and sexual maturation are inconsistent. The boars in the treatment with more than 12 h lighting time had a higher testosterone content in peripheral and seminal plasma than those in the natural spring lighting regime from January to March [62]. The 15L/9D treatment increased the libido score, sperm concentration, and total viable sperm in boars compared to the natural autumn lighting regime, with no effect on testicular volume [2]. Furthermore, 16L/8D could increase the plasma testosterone concentration in bucks compared to the natural spring lighting treatment [63]. The natural day length (September to February) could also increase the number of spermatozoa per ejaculate in boars compared to the natural increasing day length (March–August) [64]. However, the sperm quality of boars treated with the natural day length (August–November) was lower than that of the natural increasing day length (February–April) [65]. No differences were found in the testis weight, epididymal weight, and semen characteristics between boars under natural lighting or 15L/9D treatments [66]. The wavelength, intensity, and day length are crucial for regulating animal reproductive performance [67]. The red-light photo-stimulation can improve the overall sperm function and reproductive performance of boars [68], while wavelengths of 470 (blue), 497 (blue/green), and 525 (green) nm suppressed melatonin secretion [69]. The high light intensity suppressed the melatonin secretion of wild boars [70]. A high illumination intensity (65.7 lux) decreased semen volume and increased sperm quality in boar sperms [71]. It is well known that the photoperiod, light intensity, and light wavelength vary throughout the seasons. Thus, the discrepant lighting treatments (different in lighting duration, wavelengths, and intensity) may account for the inconsistent results in previous studies. In the present study, the SDL and LDL treatments had the same light parameters, with a wavelength of 450 nm and light intensity of 50 lux. The present results illustrate that day length may play a significant regulatory role in the growth and gonadal development of Meishan male pigs.

Melatonin is closely related to testicular development and testosterone secretion. Daily afternoon injections of melatonin (100 μg/day) inhibited the plasma testosterone concentration and testis weight of young male rats [72]. The implantation of melatonin (200 ng/day) in the hypothalamus reduced testis weights in mice by 60% compared to control mice [73]. Melatonin (from 10 pM to 1 μM) significantly reduced testosterone content in isolated Leydig cells from active testes of 14L/10D-treated adult hamsters but had no effect on the isolated Leydig cells from pigs who underwent the 6L/18D treatment [74]. The implantation of melatonin (18 mg) increased the mean value, basal level, and number of peaks in testosterone in rams [75]. This indicates that the differential regulatory effect of melatonin on testosterone secretion and testicular development may be related to factors such as the administration method, dosage, species, and duration of action.

Melatonin exerts its reproductive and endocrine action through the receptors MT1 and MT2 [76,77]. In rats, melatonin may also regulate testicular development directly by binding to MT1/MT2 in the testes [78]. Researchers identified that MT1 and MT2 are located in the sperm plasma membrane of boar [79]. In the present study, the boar’s testis expressed MT1 and MT2, and the MT2 protein level in the LDL group was significantly higher than in the SDL group. Tast et al. (2001) proved that pigs exhibited a precise circadian rhythm in plasma melatonin, with peak levels at the scotophase and valley levels at lighting time, and the 16L/8D-treated pigs had a shorter duration of melatonin peak level than those that underwent the 8L/16D treatment [80]. This suggests that the day length may determine the daily volume of melatonin secretion, which may consequently regulate the MT2 protein level in the testis and influence the development and function of the testis. However, further study could elucidate the interaction between melatonin and melatonin receptors.

Consistent with the positive effects of LDL treatment on the testis growth and development of testosterone secretion, LDL treatment significantly increased steroidogenesis protein levels, including SF1 and CYP19 proteins, compared to the SDL treatment. SF1 is essential for the development and function of the reproductive system and steroidogenesis-related gene transcription [81,82,83]. The disruption of SF1 usually causes testicular dysgenesis, a reduction in androgen production, and male factor infertility [84]. The pituitary-specific SF1 knockout mice exhibited severe gonadal hypoplasia [85], and the gonads were utterly absent in male newborn SF1 knockout mice [86,87]. CYP19 responses to aromatase regulation in gonad differentiation and development, and the activity of P450arom (encoding by the CYP19 gene), boost the testes’ development [88]. CYP19 knockout male mice have testes with a normal morphology, which display grossly dysmorphic seminiferous tubules, disruption spermatogenesis, a reduction in testis weight [89], and severe impairment in coital behaviors [90]. Thus, the increase in SF1 and CYP19 in the testis may account for the improvement in testis growth and testosterone secretion in the LDL-treated boars compared to the SDL-treated male pigs. In addition, previous studies showed that melatonin increased testosterone production via upregulating the SF1 expression in mammalian Leydig cells [91]. Melatonin upregulated aromatase (CYP19) expression in primary cultured human granulosa–lutein (hGL) cells through the melatonin receptor (MT1 and MT2) [92]. These suggest that the effect of day length on the abundance of MT2, CYP19, and SF1 proteins in the testis may occur via melatonin. However, further studies are needed to elucidate the effect of day length on melatonin secretion and the role of melatonin in mediating the influence of circadian rhythm on the HPT axis’ activity.

## 5. Conclusions

The present study illustrated that day length could influence the growth, gonadal development, testosterone secretion, and behaviors in Meishan male pigs, and LDL-treated Meishan male pigs had a better body size, testicular size, and testosterone content, and spent more time lying and less time exploring, due to changes in the contents of MT2, SF1, and CYP19 proteins in the testis. The present observations suggest that the LDL could be beneficial for the development of male pigs. Therefore, a proper day length should be considered in male pig breeding.

## Figures and Tables

**Table 1:** Types and definitions of boar behavioral observations.

Behaviors	Descriptions
Mounting	One pig gradually approached another pig, and then placed its foreleg span on it.
Drinking	Drinking with its mouth over drinker nozzle.
Feeding	Mouth makes contact with trough for feeding or chewing feed.
Exploring	Walking with a slow regular gait and sniffing, nosing, chewing the floor and walls of pen.
Lying	Body weight supported by belly (sternum in contact with floor) or side (shoulder in contact with floor).

**Table 2:** Body size and testicular traits.

Items	SDL	LDL	*p*-Value
Body size ^a^			
Body height (cm)	46.57 ± 1.17	51.21 ± 1.02	0.025
Body length (cm)	87.00 ± 1.60	88.50 ± 1.26	0.357
Chest circumference (cm)	75.00 ± 1.55	79.64 ± 1.53	0.011
Testicular traits ^b^			
Testicular length (mm)	89.31 ± 8.55	98.03 ± 10.03	0.033
Testicular width (mm)	51.30 ± 1.31	55.07 ± 2.21	0.133
Testicular weight (g)	96.61 ± 6.19	115.43 ± 6.81	0.034
Epididymal weight (g)	28.79 ± 3.44	29.63 ± 1.87	0.732

SDL, short day length (10L:14D); LDL, long day length (14L:10D); all the differences were considered significant at *p* < 0.05, ^a^ n = 7, ^b^ n = 4.

**Table 3:** Growth performance and digestibility.

Items	SDL	LDL	*p*-Value
Initial BW (kg)	13.36 ± 1.09	13.29 ± 1.09	0.136
Final BW (kg)	41.60 ± 3.03	45.96 ± 3.20	0.343
BWG (kg)	28.24 ± 2.45	32.67 ± 2.52	0.125
ATTD of DM (%)	74.78 ± 0.39	74.97 ± 0.59	0.800
ATTD of CP (%)	76.08 ± 2.09	84.74 ± 1.96	0.028

SDL, short day length (10L:14D); LDL, long day length (14L:10D), BW, body weight; BWG, body weight gain; ATTD, apparent total tract digestibility; DM, dry matter; CP, crude protein; all the differences were considered significant at *p* < 0.05, n = 7.

**Table 4:** Pig behavior.

Behaviors	SDL	LDL	*p*-Value
Mounting frequency (times/24 h)	7.57 ± 2.89	7.29 ± 1.71	0.918
Drinking frequency (times/24 h)	9.00 ± 2.26	10.43 ± 3.02	0.652
Feeding frequency (times/24 h)	10.00 ± 1.07	8.00 ± 1.86	0.348
Exploring time (mins/24 h)	416.29 ± 56.89	256.57 ± 32.66	0.039
Lying time (mins/24 h)	891.43 ± 33.66	1105.29 ± 31.58	0.002

The behavior of each boar over 24 h at the 12th wk was recorded and stored, and their frequency and duration were analyzed. SDL, short day length (10L:14D); LDL, long day length (14L:10D), all the differences were considered significant at *p* < 0.05, n = 7.

**Table 5:** The correlation between the fecal testosterone level, testicular weight, testicular length, and testicular width.

Items	Testosterone	Testicular Weight	Testicular Length	Testicular Width
Testosterone	r = 1	r = 0.691 *p* = 0.058	r = 0.707 *p* = 0.049	r = 0.303 *p* = 0.466
Testicular weight		r = 1	r = 0.508 *p* = 0.199	r = 0.691 *p* = 0.058
Testicular length			r = 1	r = 0.233 *p* = 0.578
Testicular width				r = 1

All the differences were considered significant at *p* < 0.05, n = 4.

## Data Availability

The data supporting the findings of this study are available within the article, there is no undisclosed data in this study.

## Supplementary Materials

The following supporting information can be downloaded at: https://www.mdpi.com/article/10.3390/ani14060876/s1, Table S1: The cross-reactivity of every antibody. Referencecs [93,94,95,96,97] are cited in the Supplementary Materials File.
