# Supplementary material for: Effect of Day Length on Growth and Gonadal Development in Meishan Male Pigs
Source: Animals (Basel). 2024 Mar 13;14(6):876. doi: 10.3390/ani14060876 (PMC10967555; doi:10.3390/ani14060876)
Supplement: Supplementary file 1 [file animals-14-00876-s001.zip › animals-2857979-supplementary.pdf]

Table S1. The cross reactivity of every antibody.

**Protein symbol**	**Product No.**	**Source**	**Cross reactivity**
β-actin	bs-0061R	Rabbit	Human, Mouse, Rat, Hamster (Predicted: Rabbit, **Pig**, Sheep, Chicken, Dog, Cat, GuineaPig, Fish, Bee)
MT1	bs-0027R	Rabbit	Human, Mouse, Rat (Predicted: **Pig**, Sheep, Cow, Chicken, Dog, GuineaPig)
MT2	bs-0963R	Rabbit	Human, Mouse, Rat (Predicted: Dog) (Homology with pigs: 80.06%)
SF1	sc-28740	Rabbit	Mouse, Rat, Human, Equine, Canine, Bovine, **Porcine**
StAR	sc-25806	Rabbit	Mouse, Rat, Human, Equine, Bovine, **Porcine**
3β-HSD	sc-30820	Goat	Mouse, Rat, Human, Equine, Canine, Bovine, **Porcine**
P450scc	sc-18040	Goat	Human, Mouse, Rat, Canine (Homology with pigs: 79.04%)
CYP19	sc-374176	Mouse	Human (Homology with pigs: 83.89%)

Notes: The β-actin, MT1, MT2, SF1, StAR, 3β-HSD and P450scc antibodies used in this study had been successfully tested in pig sperm and granulosa cells samples [1,2]. And the CYP19 antibody used in this study had been successfully tested in mouse granulosa cells [3] (Homology with mouse: 79.32%) , horse testis [4] (Homology with horse: 78.33%) and fish testis samples [5] (Homology with fish: 54.08%).

References:

1. Lu N, Li M, Lei H, Jiang X, Tu W, Lu Y, Xia D. Butyric acid regulates progesterone and estradiol secretion via cAMP signaling pathway in porcine granulosa cells. J Steroid Biochem Mol Biol. 2017;172:89-97.
2. Lu N, Jiang X, Zhang C, Li B, Tu W, Lei H, Yao W, Xia D. Melatonin mediates via melatonin receptor 1 in a temperature-dependent manner regulating ATP metabolism and antioxidative enzyme activity of boar spermatozoa in vitro. Theriogenology. 2022;188:1-12.
3. Hu J, Jin J, Qu Y, Liu W, Ma Z, Zhang J, Chen F. ERO1α inhibits cell apoptosis and regulates steroidogenesis in mouse granulosa cells. Mol Cell Endocrinol. 2020;511:110842.
4. Rouge M, Legendre F, Elkhatib R, Delalande C, Cognié J, Reigner F, Barrière P, Deleuze S, Hanoux V, Galéra P, Bouraïma-Lelong H. Early Castration in Horses Does Not Impact Osteoarticular Metabolism. Int J Mol Sci. 2023;24:16778.
5. Santos D, Rocha E, Malhão F, Lopes C, Gonçalves JF, Madureira TV. Multi-Parametric Portfolio to Assess the Fitness and Gonadal Maturation in Four Key Reproductive Phases of Brown Trout. Animals. 2021;11:1290.
